# 

**Published:** 1842-08-20

**Authors:** 


### MEDICAL EXAMINER,

EDITED BY

*J. B. BIDDLE, M.D. & W. W. GERHARD, M.D.*

Vol. I.] August 20, 1842. [No. 34.

